# Human Hazard Assessment Using *Drosophila* Wing Spot Test as an Alternative In Vivo Model for Genotoxicity Testing—A Review

**DOI:** 10.3390/ijms22189932

**Published:** 2021-09-14

**Authors:** Pornsiri Pitchakarn, Woorawee Inthachat, Jirarat Karinchai, Piya Temviriyanukul

**Affiliations:** 1 Department of Biochemistry, Faculty of Medicine, Chiang Mai University, Chiang Mai 50200, Thailand; pornsiri.p@cmu.ac.th (P.P.); jirarat.ka@cmu.ac.th (J.K.); 2 Food and Nutrition Academic and Research Cluster, Institute of Nutrition, Mahidol University, Salaya, Phuttamonthon, Nakhon Pathom 73170, Thailand; woorawee.int@mahidol.ac.th

**Keywords:** *Drosophila melanogaster*, carcinogens, genotoxicity testing, human hazard assessment, mutagens, wing spot test

## Abstract

Genomic instability, one of cancer’s hallmarks, is induced by genotoxins from endogenous and exogenous sources, including reactive oxygen species (ROS), diet, and environmental pollutants. A sensitive in vivo genotoxicity test is required for the identification of human hazards to reduce the potential health risk. The somatic mutation and recombination test (SMART) or wing spot test is a genotoxicity assay involving *Drosophila melanogaster* (fruit fly) as a classical, alternative human model. This review describes the principle of the SMART assay in conjunction with its advantages and disadvantages and discusses applications of the assay covering all segments of health-related industries, including food, dietary supplements, drug industries, pesticides, and herbicides, as well as nanoparticles. Chemopreventive strategies are outlined as a global health trend for the anti-genotoxicity of interesting herbal extract compounds determined by SMART assay. The successful application of *Drosophila* for high-throughput screening of mutagens is also discussed as a future perspective.

## 1. Introduction

**Figure 1:**
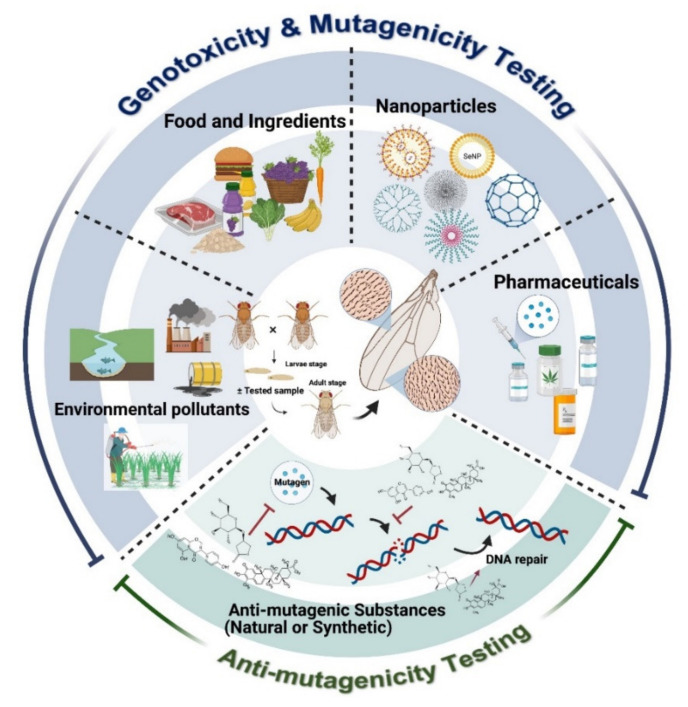
Applications of SMART or wing spot test in human hazard assessment covering food and drug safety, nanoparticles, pesticide and herbicide, and environmental safety. SMART is also applied to test anti-mutagenicity properties of the interested agents.

Maintaining a stable genome is of the greatest importance for the proper functioning of life. DNA damages, induced by mutagens or genotoxins, lead to genomic instability that is strongly associated with premature aging [1,2,3] and, eventually, carcinogenesis [4,5]. Maurici et al. defined the difference between mutagens and genotoxins. A mutagen is a chemical that induces permanent and transmissible changes in a gene(s). This event is called “mutagenicity”, while genotoxicity refers to events where chemicals (genotoxins) induce changes not only in DNA sequences but also in cellular compartments involving genome integrity [6]. Mutagen/genotoxic agents can be divided into two groups as (i) endogenous genotoxins such as reactive oxygen species (ROS) that oxidize guanine, leading to the formation of the most common DNA adducts, 8-oxoguanine (8-oxoG) [7]. These highly mutagenic lesions can pair to either cytosine (C) or adenine (A) in the DNA leading to T-to-G mutations [8]. Another source is (ii) exogenous genotoxins, including those present in food, medicine, cosmetics, air pollution, radiation, and sunlight. Unfortunately, in some cases, exposure to genotoxic agents from either endogenous or exogenous sources is unavoidable. Therefore, an in vivo assay equipped with high reliability is necessary to maximize genome safety and cancer prevention. The wing spot test or somatic mutation and recombination test (SMART) involving *Drosophila melanogaster* (fruit fly) is a short-term test and an alternative non-mammalian system for in vivo testing of suspected genotoxins present in the environment. *D. melanogaster* has a relatively short life span, rendering quick reproductive cycles and high-throughput genotoxicity screening. *D. melanogaster* also harbors functional orthologs of human disease-related genes at about 75%, making it an ideal in vivo model for human study [9]. This review summarizes the basic principle of the SMART assay and its application, from past to present, in human hazard assessment covering food and drug safety, nanoparticles, pesticide and herbicide assessment, and environmental safety. The application of *D. melanogaster* as a model to evaluate the anti-mutagenicity of chemical compounds and high-throughput screening of mutagens using SMART is also discussed (Figure 1).

## 2. Principle of the SMART Assay or Wing Spot Test

The somatic mutation and recombination test (SMART) or wing spot test relies on the genetic damage induction of dividing wing disc cells, resulting in loss of heterozygosity (LOH) during larval development, which can be obviously seen on the adult wings as mutant wing spots [10]. The imaginal discs are epithelial structures that develop in the fly larva and constitute the precursors of the adult cuticle of the flies. There are a total of nineteen imaginal discs in the larva [11,12], each of which is named for the adult structure it forms, for instance, the eye-antennal discs are responsible for the development of compound eye and the antenna in adults, while the wing imaginal discs give rise to the wing and thorax. The imaginal disc cells can duplicate every 10 h during larval development, indicating their rapid proliferation rate [11]. Because of the rapid cell proliferation, genotoxins have a greater chance of interacting with the larval genome, rendering SMART a sensitive genotoxicity test.

**Figure 2:**
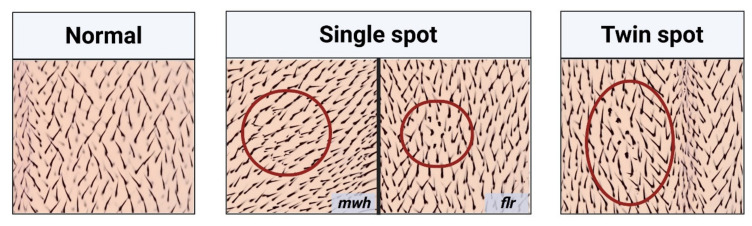
Characteristics observed on the *Drosophila* wings. (Left) Characteristics of normal wing spots (each hair in the adult wing is the result of the cuticular secretion of each wing disc cell); (Middle and Right) characteristics of mutant wing spots (single spots (middle panel) and twin spots (right panel)), which can be induced by genotoxins. *mwh*— multiple wing hairs, and *flr*—flare.

Genotoxins that cause point mutations, DNA or chromosome breaks, chromosome rearrangement, as well as mitotic recombination in the larval wing disc cells produce abnormal hair spots or mutant spots on the wings of the tested flies as single spots and twin spots (Figure 2) [10], stating the advantage of the SMART assay by detecting wide ranges of genotoxins [13]. Furthermore, during the experiment, the larvae are fed by the tested compounds that are processed or metabolized in the same way as they are consumed [14]. The SMART assay is, therefore, suitable for testing the genotoxic potential of single or complex compounds, food, beverage, herbal extracts, and even polluted water. 

**Figure 3:**
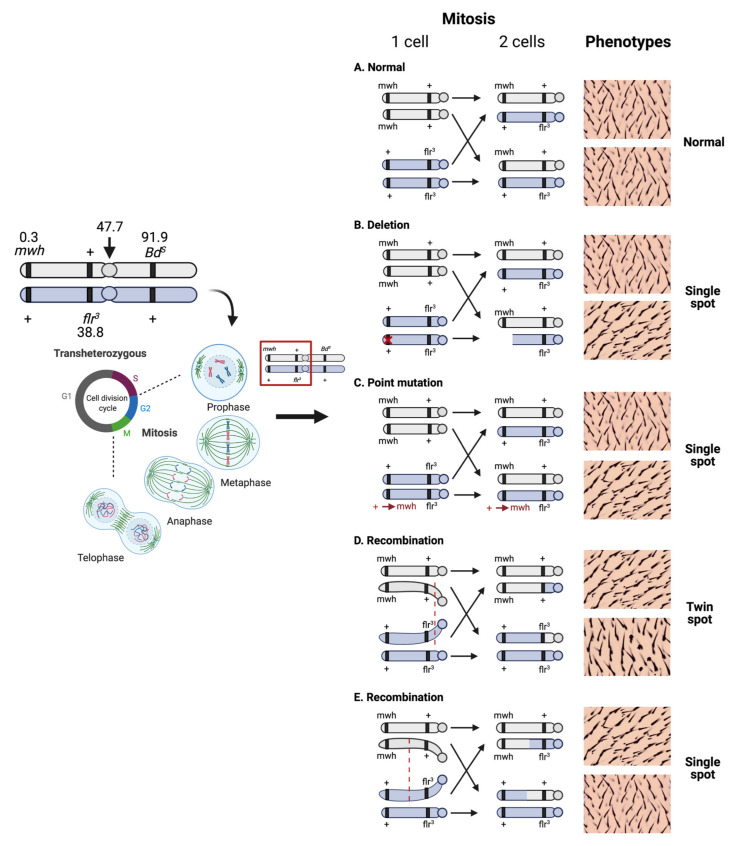
The schematic diagram illustrating the molecular mechanisms for mutant spots (single or twin spots) formation by deletion, point mutation, and recombination detected by wing spot test (adapted from [10]).

The SMART assay makes use of three *Drosophila* strains, which are (i) multiple wing hairs (*mwh*/*mwh*); (ii) *flare* (*flr^3^/In(3LR) TM3*, *ri p^p^ sep l(3)89Aa bx^34e^ e Bd^S^*); and (iii) Oregon-*flare* (*ORR/ORR;flr^3^/In(3LR) TM3, ri p^p^ sep l(3)89Aa bx^34e^ e Bd^S^*) [13,15]. More information on the genetic symbols and descriptions was defined by Lindsley and Zimm [16]. The mwh marker is located on chromosome 3, and its unique phenotype is multiple trichomes per cell when homozygous. The *flare* marker is recessive and also located on chromosome 3. This produces abnormal epidermal point-like hairs on the wing [17]. The Oregon-*flare* exhibits the *flare* phenotype as described; however, its chromosomes 1 and 2 are from DDT-resistant Oregon R(R), which harbors a high constitutive expression of cytochrome P450 [17,18,19], a major Phase I enzyme in the xenobiotic bioactivation system. Therefore, the cross between (i) and (ii) generates trans-heterozygous larvae as a standard cross (ST cross), while mating between (i) and (iii) produces trans-heterozygous larvae possessing higher metabolic activity of Phase I enzyme than normal to activate pro-mutagens as an improved high bioactivation (HB) cross. Performing both the ST and HB cross during genotoxicity evaluation of suspected compounds offers a useful tool to determine the in vivo potential genotoxicity of compounds and also reveals the nature of either direct-acting (genotoxic effects could be observed in ST cross) or indirect-acting (genotoxic effects are observed in only HB cross) genotoxins. The schematic diagram illustrating mutant spots (single or twin spots) formation is depicted in Figure 3. According to Figure 3, the single spots result from chemical-induced point mutation or deletion, while mitotic recombination is mostly caused by the recombinogenic activity of a testing agent.

## 3. Applications of SMART for Food Safety

Food and dietary supplement industries have recently mushroomed as a result of new advanced production technologies, the increasing trend of healthy food, and the search for novel nutrients and phytochemical sources. Substances used in these industries have also increased, including food dyes, food preservatives, synthetic flavors, and aroma, modern technology-derived food, plant/herbal extracts, as well as novel food. These substances, in certain circumstances, may be important health risks. The European Food Safety Authority (EFSA) states that “any food not consumed “significantly” by humans in the EU before 15 May 1997 is considered to be a novel food”. Thereby, according to this definition, novel food can be food developed using new production technologies or processes, plant and herbal extracts, and even food that has been usually consumed outside of the EU. Thus, novel food must be subjected to food safety and genotoxicity assessment as one of the safety requirements. As such, SMART could be employed to evaluate the genotoxic potential of several food products or plant extracts used in food industries. Table 1 summarizes some applications of SMART for testing food preservatives, food dyes, synthetic flavors and aromas, plant/herbal extracts, and phytochemicals. Table 1 also indicates that performing the SMART assay should employ both ST and HB cross together with dose variation because this can help to distinguish between direct and indirect mutagens as well as safe doses. Hence, it is unclear whether food dyes and food flavors are mutagens by the SMART test since they have not yet been tested using HB cross. Interestingly, SMART data show that most food preservatives widely used in food industries have mutagenic potentials at high doses. There is a link between dietary nitrite and stomach cancer and the combination of nitrite and nitrate and colorectal cancer [20].

## 4. Applications of SMART in Drug Safety Assessment

Drug discovery and development is a process to deliver safe and effective drugs. A new generation of modern drugs with reduced toxicity and enhanced efficacy has been discovered and developed to replace traditional drugs. Drug safety evaluation, as one of five steps in the drug development process according to the FDA, provides information for both consumers and health professionals [43].

The SMART assay is a practical test for potential chronic use and overdosing genotoxic property assessment of pharmaceutical agents [44,45]. Several studies confirmed SMART as a suitable in vivo test to evaluate the genotoxicity of anesthetic agents. For example, ketamine and rocuronium bromide, common anesthetic agents, were subjected to the wing spot test. Results showed that ketamine was genotoxic in both the standard (ST) cross (250 μg/mL) and the high-bioactivation (HB) cross (1000 μg/mL), while rocuronium bromide exerted genotoxic effects exclusively in HB cross. Moreover, after evidence of its severe toxicity, levobupivacaine, a new local anesthetic, has been developed as an alternative to bupivacaine. Data from standard crosses showed that bupivacaine and levobupivacaine were not mutagenic or recombinogenic agents. Levobupivacaine was genome safe even at high doses (1000 µg/mL) in the HB cross [46]. Thus, these studies showed that SMART contributed to the genotoxicity assessment of the anesthetic agents.

SMART can also be used to evaluate the long-term genotoxicity of oral medicines. Long-term treatment of sulfonylureas caused micronucleus formation, a marker for DNA breaks, in type 2 diabetes mellitus (T2DM) patients [47]. Gürbüzel et al. [44] investigated two sulfonylureas as glimepiride and glipizide for their genotoxicity using SMART. Both sulfonylureas displayed genotoxic effects, possibly due to homologous somatic recombination [44]. Their results increased the awareness of using sulfonylureas as T2DM medicines. Another genotoxicity study investigated the selective serotonin reuptake inhibitors (SSRIs) citalopram and sertraline. Findings from SMART showed that citalopram could act as a genotoxin, while sertraline was devoid of genotoxic properties. SSRIs are prescribed to treat depression as long-term use, with increased chronic exposure and health risks [48]. The wing spot test or SMART has the advantage of being quick and inexpensive to assess drug safety. Table 2 summarizes the applications of SMART for testing some medicines.

## 5. Applications of SMART for Genotoxicity Assessment of Environmental Pollutants

Humans are exposed to a range of substances that may cause acute or chronic toxic, genotoxic, or carcinogenic hazards. The environment is polluted with a large range of compounds that are commonly used as pesticides in industries or agriculture. Nowadays, the public show increased concern regarding chemical contamination. Long-term exposure to pollutants presents in the environment and foods can adversely impact human health by disturbing biochemical and physiological pathways or genome stability. Recent evidence suggests a relationship between pollutant exposure and the development of chronic non-communicable diseases (NCDs), including cancers, T2DM, congenital malformations, and degenerative diseases [52,53]. Evaluating the impact of extended exposure to environmental pollutants at low concentration on human health is difficult because the symptoms are often not clinically apparent [54]. The information available regarding their toxicity is not sufficient to anticipate the occupational danger since certain compositions may include hazardous chemicals that operate at the molecular level and can cause genotoxic and mutagenic consequences [55]. Additionally, DNA alterations may result in mutation retentions that, if left uncorrected, may accumulate and initiate malignant processes [56]. This section demonstrates the usefulness of the SMART assay to assess genotoxicity in the wings of *Drosophila* exposed to environmental pollutants. SMART assay results can be used to build up a more comprehensive database on the genotoxicity of many environmental pollutants, leading to additional research stating the genotoxicity of identified genotoxic substances and their adverse effects on human health and the environment.

### 5.1. Insecticides and Herbicides

Providing adequate food for the growing global population is a significant challenge. Agricultural land area is reducing through urbanization, industrialization, and soil erosion, while more than 1500 chemicals are licensed for use as pesticides and discharged into the environment. Pesticides have a number of unintended consequences that include poisoning of humans and animals and the development of pesticide resistance.

Pyrethroids are a group of synthetic organic compounds that are widely used as commercial household insecticides to control insects and mosquitoes, with subsequent long-term usage risks. Fast-acting pyrethroid insecticides contain transfluthrin or benfluthrin as volatile compounds. These contact and inhalation agents are used as household hygiene products against flying insects, including mosquitoes, flies, moths, and cockroaches. Another pyrethroid, metofluthrin, is particularly vapor-active against insects and mosquitoes for both indoor and outdoor use [57,58]. Pyrethroids are neurotoxic, possibly by disturbing the membrane function of neuronal cells [59]. The possible genotoxicity of transfluthrin and metofluthrin was detected by SMART, with transfluthrin (up to 0.103 mg/mL) and metofluthrin (up to 60 μg/mL) as a mutagen and recombinogen, respectively. 

Another commercial insect repellent is fipronil which belongs to the phenylpyrazole family. Fibronil kills insects via impaired nerve impulse, increased excessive neuronal activity, and paralysis [60]. Fibronil is considered toxic to humans and the environment and has been reported for toxicity against non-target organisms [61]. The SMART assay was employed to determine the genotoxicity of fibronil. Data showed that all tested concentrations of fibronil (0.3, 0.7, 1.5, and 3.0 × 10^−5^ mM) were positive in both ST and HB cross, indicating its strong mutagenic and recombinogenic properties [62]. The tumorigenesis effect of fibronil in flies was also tested. Data confirmed that as well as having mutagenic and recombinogenic properties, fibronil was carcinogenic and induced epithelial tumor frequency. Taken together, data from *Drosophila* revealed that the insect repellent, fibronil, was mutagenic, recombinogenic, and carcinogenic, necessitating exposure awareness.

Thiamethoxam is currently the most commercialized insecticide worldwide [63] as a neonicotinoid targeting the insect neuronal system. Neonicotinoids are used to control pests such as ants, aphids, whiteflies, beetles, and some lepidopterans [64]. The advantage of thiamethoxam over other insecticides such as carbamate, pyrethroid, and organophosphate is its selective toxicity against insects [65]. Reports showed that neonicotinoids could cause DNA damage [66]; however, genotoxicity data of thiamethoxam was missing. de Morais et al. (2017) evaluated the genotoxicity of thiamethoxam at concentrations from 2.4 × 10^−4^ to 1.9 × 10^−3^ mM. Results showed that all tested doses were not mutagenic in the ST cross, while high doses (from 9.7 × 10^−4^ to 1.9 × 10^−3^ mM) were mutagenic in the HB cross, suggesting that thiamethoxam was a promutagen and its metabolites caused DNA damage [57]. Results showed that *Drosophila* was a promising in vivo model organism for the testing of genotoxicity of insect repellents and sensitive enough to distinguish between direct and indirect mutagens.

In addition to insecticides, herbicides are also commonly used in agriculture to control undesirable vegetation. Many herbicides are not genotoxic but exhibit DNA damaging effects after biotransformation in the body. As mentioned, SMART is an in vivo system mimicking biotransformation similar to that of mammals [67]. Table 3 summarizes the applications of SMART for testing insecticides and herbicides. Interestingly, most herbicides were subjected to genotoxicity testing using SMART compared to insecticides, possibly due to controversial results on the genotoxic effects of widely used herbicides such as 2,4,5-trichlorophenoxyacetic acid (2,4,5-T), bentazone, glyphosate, molinate, and trifluralin. For instance, 2,4,5-T, a chlorinated phenoxy acetic acid as a hormone herbicide, was mutagenic in *S. cerevisiae* [68], whereas it was not mutagenic in the bacterial assay (Ames test) [69]. However, with advantages over yeast and bacteria, results from SMART demonstrated that 2,4,5-T induced small single spots on the wing but not in the other types of abnormal clones, particularly the ST cross. Thus, the SMART data added new information regarding the genotoxic effect of 2,4,5-T. Genotoxicity data from SMART showed that most herbicides were direct-acting genotoxins (positive in ST cross), including glyphosate, 2,4,5-T, and thiobencarb. Intriguingly, they were not mutagenic after biotransformation, implying that metabolic activation by *Drosophila* Phase I and Phase II enzymes suppressed the mutagenic potential of some herbicides.

### 5.2. Chemicals in Daily Life

This section presents examples of using SMART for genotoxicity assessment of chemicals in daily life, including polycyclic aromatic hydrocarbons (PAHs), compounds occurring during combustion, parabens, naphthalene, 1-nitronaphthalene, and 1,5-dinitronaphthalene. Table 4 summarizes the applications of SMART for testing chemicals in daily life.

PAHs are organic compounds known to exert genotoxic effects [75]. PAHs can be generated during incomplete natural combustion or man-made combustion sources such as tobacco smoke and combustion of biofuels. PAHs are widespread in the environment and found in air, soil, and water [76]. Around one thousand tons of PAHs are emitted into the atmosphere each year [77] as a potential human health risk. SMART was used to test the genotoxicity of PAHs. Results showed that PAHs and their nitro derivatives were genotoxic. Interestingly, PAH derivatives exhibited different patterns of genotoxicity. For instance, naphthalene, 1-nitronaphthalene, and 1,5-dinitronaphthalene were more genotoxic after metabolic activation (more positive in HB cross than ST cross), while anthracene was positive only in HB cross [78], possibly due to the characteristics, chemical properties, and metabolism of each PAH compound [79]. Moreover, the SMART assay suggested that genotoxicity of PAHs occurred primarily after metabolic activation [80].

Nitrates are other chemicals with human exposure through consumption of leafy green vegetables, cured meat, and drinking water. After ingestion, nitrate is converted into nitrite, which subsequently interacts with amines and amides to form N-nitroso compounds in the gastrointestinal tract. N-nitroso compounds (NOCs) are classified as carcinogenic in humans. Sodium nitrite is commonly used as a food preservative and antimicrobial agent in food industries; thus, it poses a genotoxic risk to humans. The SMART assay showed that sodium nitrite and sodium nitrate were able to induce somatic mutation and recombination in a dose-dependent manner [23]. SMART data revealed that sodium nitrite was converted into sodium nitrate in the *Drosophila* digestive tract in a similar way to mammals, suggesting that the wing spot test can be widely utilized as an in vivo assay indicator to monitor nitrate and nitrite genotoxicity in humans.

Parabens are a group of preservative ingredients broadly used in the cosmetic and drug industries, with long-term exposure to humans. Some controversial reports were presented on paraben toxicity [80,81]. Methylparaben and propylparaben were subjected to genotoxicity analysis by SMART. Results showed that methylparaben and propylparaben were not mutagenic at the tested doses (100–250 mM) in ST cross [82]. However, a clear conclusion is awaited since there is no current study in HB cross.

Other chemicals that should be discussed include bisphenol A (BPA). BPA is a precursor of plastic production and epoxy resin used as food packaging material [83]. BPA can be contaminated into the human body through food and water consumption, inhalation, or skin contact. BPA can also be released during dishwashing, boiling, and brushing into the environment [84]. Estimates suggest that one hundred tons of BPA pollute the environment annually [85,86].

Several scientific studies in humans have shown a link between BPA exposure and a variety of illnesses such as diabetes, obesity, cardiovascular disease, and cancer [87,88]. However, the genotoxicity data of BPA remain unclear [89,90]. BPA was subjected to genotoxicity analysis using the wing spot test. Results revealed that BPA significantly induced single spot mutants in both ST and HB crosses, but inconclusive results were unclear in total spot mutants for both tested strains. The data implied that BPA might act as a direct-acting mutagen, leading to point mutation rather than recombination [91].

**Table 4:** Applications of SMART for testing chemicals in daily life.

Types	Result in ST Cross	Result in HB Cross	Range of Treated Doses and Remarks	Ref.
1,5-Dinitronaphthalene	+	+	1–100 mM, inconclusive at 20 and 50 mM and positive at 1, 10, and 100 mM positive in ST cross; however, all doses positive in HT cross	[78]
2-Methylisoborneol	-	nd	125–500 µg/mL, all doses lack genotoxicity	[92]
4-(methylnitrosamino)-1-(3-pyridyl)-1-butanone (NNK)	+	+	0.6–2.4 mM, all doses positive both ST and HB cross	[93]
1-Nitronaphthalene	+	+	1–5 mM, lack genotoxicity at 1 mM and positive at 2–5 mM in ST cross, all doses positive in HB cross	[78]
9-Nitroanthracene	+	+	1–20 mM, inconclusive up to 10 mM, and positive at 20 mM in ST cross; however, lack of genotoxicity at 5 mM, inconclusive at 20 mM and positive at 1, 10, and 50 mM in HB cross	[78]
α-Terpineol	-	-	0.05 and 2.5 µg/mL, lack genotoxicity at 0.05 µg/mL and inconclusive at 2.5 µg/mL in ST cross; however, lack genotoxicity in HB cross	[94]
Anthracene	+	+	1–50 mM, lack genotoxicity at 5, 20, and 50 mM and positive at 1 and 10 mM in ST cross, HB cross shows 50 mM lack genotoxicity, weak positive at 1 and 10 mM, and positive at 5 and 20 mM	[78]
Bisphenol A	-	-	0.1–5 µg/mL, all doses show inconclusive genotoxicity both ST and HB cross	[91]
Dimethylarsinic acid	+	nd	0.05–0.5 mM, lack of genotoxicity at 0.1 mM but 0.05 mM inconclusive and positive at 0.25 and 0.5 mM	[95]
Linalool	-	-	0.025 and 2.5 µg/mL, inconclusive at 0.025 µg/mL and lack genotoxicity at 2.5 µg/mL in ST cross; however, lack genotoxicity HB cross	[94]
Methylparaben	-	nd	100–250 mM, all doses lack genotoxicity	[82]
Naphthalene	+	+	1–10 mM, inconclusive at 1 mM and positive at 5 and 10 mM in ST cross; moreover, weak positive at 1mM and positive at 5 and 10 mM in HB cross	[78]
N-Nitrosonornicotine (NNN)	+	+	7.2–28.8 mM, all doses positive both ST and HB cross	[93]
Propylparaben	-	nd	100–250 mM, lack of genotoxicity up to 150 mM and inconclusive at 200 and 250 mM	[82]
Trans-pinocarveol	-	-	0.025 and 2.5 µg/mL, inconclusive both concentrations in ST cross; however, lack genotoxicity HB cross	[94]
Verbenone	-	-	0.05 and 2.5 µg/mL, inconclusive at 0.05 µg/mL and lack of genotoxicity at 2.5 µg/mL in ST cross; all doses lack genotoxicity in HB cross	[94]

nd—not determined, (-)—negative, and (+)—positive.

### 5.3. Domestic and Industrial Sewage

This section summarizes the applications of SMART in monitoring genotoxic, mutagenic, and recombinogenic potential in the aquatic environment and, consequently, water quality or ecological health [96]. Instead of monitoring water contamination, monitoring of water quality by SMART may provide additional safety for public health. River water samples should be collected at various time points and areas to reduce variations. The genotoxins in the samples were combined as a mixture and require further identification; however, the SMART or wing spot test enables a rapid and reliable assay to check wastewater quality. Examples of identified contaminants in river water include phosphorus and nitrogen that allow cyanobacteria blooms. These bacteria generate and release the metabolite 2-methylisoborneol (2-MIB) in the river [93]. The compound did not induce genome instability detected by SMART. Another study found that the presence of heavy metals such as lead (Pb) and cadmium (Cd) or inorganic elements, especially aluminum, silicon, sulfur, titanium, and zinc in the river, caused a significant rise in SMART [97]. Data from toxicological genetic assays assessed by SMART can be used to monitor and demonstrate whether environmental parameters are altered, thereby resulting in deleterious conditions. Table 5 summarizes the applications of SMART for testing wastewater.

## 6. Applications of SMART for Nanoparticle Genotoxicity Assessment

Nanomaterials with intriguing physicochemical characteristics are commercially accessible for a variety of applications, including cosmetics, medicine, and functional foods. These materials may spread into the environment, leading to human exposure. Thus, it is critical to observe whether these nanoparticles have mutagenic effects because they can be easily ingested and incorporated into various tissues. *Drosophila* remains a viable alternative model for nanoparticle toxicity assessment to prevent or minimize the usage of mammals [102]. The wing spot test has been used to investigate the mutagenic potential of nanoparticles to ensure their safe use [103,104,105]. Results from SMART showed that the genotoxicity of each type of nanoparticle depended on several factors such as size, form, dose, and type of fabrication.

Previous studies found that low concentrations of cobalt nanoparticles (Co-NPs) were more genotoxic than cobalt chloride, whereas high doses of cobalt chloride (10 mM) exhibited a higher frequency of total spots compared to Co-NPs [106], possibly due to the cellular uptake capacity of each form of cobalt. By contrast, some types of nanoparticles showed less toxicity than bulk materials [107], whereas nickel-based nanoparticles (NiO-NPs) [108] and copper oxide nanoparticles (CuO-NPs) [105] caused more active genotoxicity in *Drosophila* than their naïve forms that may be mediated by oxidative stress. Some studies also reported the use of SMART to assess the genotoxicity of various types of carbon nanotubes (CNTs), including single-walled carbon nanotubes (SWNTs), double-walled carbon nanotubes (DWCNTs), and multi-walled carbon nanotubes (MWNTs) [109]. Carbon nanotubes are employed as nanocarriers for proteins, nucleic acids, and bioactive molecules with high selectivity [110]. Titanium dioxide nanocrystalline structures (TiO_2_ NCs) are another type of nanomaterial used in everyday life as food colorings, personal care products (toothpaste, sunscreen), and drug coating materials [111]. TiO_2_ NCs showed genotoxicity and mutagenicity when assessed by SMART [112]. Thus, the usage of these nanomaterials should be carefully scrutinized. Studies investigating the role of SMART in nanoparticle genotoxicity assessment are summarized in Table 6.

## 7. Applications of SMART in Anti-Genotoxic Studies

As previously mentioned, many chemicals used in everyday life present human health risks. Dietary and environmental mutagens, including N-nitroso compounds in food treated with sodium nitrite (NaNO_2_), mycotoxin-contaminated cereals, and acrylamide in fried potatoes, are also highly mutagenic but hard to avoid in daily life. DNA mutations correlate with carcinogenesis, while genomic instability is a well-known cancer hallmark [4,5]. Thus, minimizing the deleterious effects of genotoxic agents by anti-genotoxic compounds is a good sustainable chemopreventive strategy to reduce cancer incidents [117]. Importantly, the SMART assay or wing spot test can be utilized as a human hazard identification test to unveil the mutagenicity potential of compounds and also used to determine the anti-genotoxic properties of chemicals against mutagens and recombinogens.

Consumption of fruits and vegetables may reduce cancer risks because they contain fiber, minerals, and phytochemicals that act as anti-mutagens or anti-carcinogens [117,118] with low toxicity compared to present cancer drugs. Numerous studies have investigated the role of phytochemicals or herbal extracts against known mutagens/carcinogens using the SMART assay. These are documented and summarized in Table 7. Interestingly, results showed that many herbal extracts possess anti-genotoxic properties against various types of genotoxic agents. More details about the use of genotoxic agents are presented as a footnote under Table 7. The anti-genotoxic properties of herbal extracts result from (i) their ability to scavenge free radicals produced by genotoxins, especially hydrogen peroxide and doxorubicin [39,119], probably by induction of the antioxidant enzymes superoxide dismutase, glutathione peroxidase, and glutathione reductase; (ii) induction of phase II metabolizing enzymes leading to an enhanced excretion of genotoxins and their metabolism before interacting with DNA, for example, Noni fruit juice may act as a quinone reductase (QR) inducer [120]; and (iii) direct interaction between bioactive constituents and genotoxins, thereby preventing binding to DNA or other cellular compartments involving genome integrity. *Buddleja globosa* leaf extract might directly interact with ethyl methanesulfonate (EMS), an ethylating agent causing DNA mutation by transferring alkyl group to nucleotides, thereby inhibiting the alkylation process [121].

## 8. Perspective: High-Throughput Screening Using SMART Assay

The SMART assay or wing spot test is a useful alternative as a fast in vivo genotoxicity test for human hazard identification that can be employed across various industries, as previously mentioned. Nevertheless, one major bottleneck that limits high-throughput screening by the SMART assay compared to other genotoxicity tests such as comet assay and micronucleus assay [143,144] is the wing spot analysis. At this step, in the classical assay, the researcher counts the mutant spots under the microscope. This is a time- and labor-consuming task. To enhance the feasibility and reproducibility of the SMART assay, Lombardot et al. developed an automated readout system for high-throughput screening of SMART [145]. This reduced operating time by approximately eight folds compared to the classic SMART protocol. Automated imaging was developed together with image analysis. The authors claimed that confocal microscopy was not needed during image acquisition. A wing score-based dose-dependency approach was also developed to offer genotoxicity profiles. The developed automated SMART assay also showed promising data by distinguishing between non-genotoxins (isoniazid, antipyrine, and atenolol) and genotoxins (mitomycin C, methylmethane sulphonate, and urethane), similar to the classical SMART assay. This development of the automated SMART assay showed promise, but some issues still required improvement. In the classical wing spot test, the *mwh* phenotype is scored as well as the *flr* phenotype. However, the developed automated analysis only detected the *mwh* phenotype [145]. The development of image analysis covering both *mwh* and *flr* phenotypes would increase the assay sensitivity. Mutant spots near the wing base were excluded from the analysis. Including this area might increase the precision of the assay.

## 9. Limitations of SMART

The Organization for Economic Co-operation and Development (OECD) provides various guidelines for genetic toxicity testing, for example, Test No. 471: bacterial reverse mutation test (Ames test) [146], Test No. 474: mammalian erythrocyte micronucleus test [147], Test No. 487: in vitro mammalian cell micronucleus test [148], and Test No. 489: in vivo mammalian alkaline comet assay [149], suggesting there is no best technique for determining genotoxicity. The Ames test can detect various types of mutagens, albeit at least five bacterial strains are recommended, and liver homogenate is needed for covering both direct and indirect-acting mutagens, while alkaline comet assay detects single- and double-stranded breaks, which might lead to chromosome damages. The chromosome damages can subsequently be detected by the micronucleus (MN) test. Interestingly, SMART assay can detect most types of DNA damages, as mentioned earlier. However, its limitation depends on the fixed developmental time restricting the exposure time to the chemical of interest. Thus, chronic exposure to toxins is the barrier, comparable to all in vitro genotoxicity testing, such as the Ames test. Moreover, wing scoring might be labor intensive compared to comet tail or MN scoring. Furthermore, in mammals, the phosphorylation of histone variant H2AX at serine 139 (γ-H2AX) has been used as a DNA damage marker [150]. In flies, histone variant H2Av is phosphorylated in response to DNA damages as well, suggesting the functional homolog between human H2AX and *Drosophila* H2Av. However, the phosphorylation of histone variant H2AX or H2Av is associated with the formation of double-stranded breaks [151], implying its limited uses for some types of genotoxic agents. 

## 10. Conclusions

The evidence demonstrated that *D. melanogaster* is suitable as an alternative human disease model for genotoxicity testing using the SMART assay or wing spot test. The advantages of the assay are (i) the assay is sensitive, inexpensive, and requires minimal infrastructure; (ii) the ethical approval is not complicated; (iii) SMART exhibits great reproducibility; (iv) SMART detects wide ranges of genotoxins, particularly when both ST and HB cross are used; and (v) automated high-throughput screening can facilitate the use of the SMART assay. The SMART assay is a useful alternative in vivo model for human hazard identification, covering all segments of health-related industries, including food, dietary supplements, cosmetics and drug industries, pesticides and herbicides, and nanoparticles.

## Figures and Tables

**Table 1:** Applications of SMART for testing food preservatives, food dyes, synthetic flavors and aroma, plant/herbal extracts, and phytochemicals.

Types	Result in ST Cross	Result in HB Cross	Range of Treated Doses and Remarks	Ref.
**Food preservatives**				
Benzaldehyde (C_6_H_5_CHO)	+	nd	0.1–50 mM, inconclusive up to 0.5 mM and positive at 1–50 mM	[21]
Benzyl acetate (CH_3_COOCH_2_C_6_H_5_)	+	nd	0.1–50 mM, lack of genotoxicity or inconclusive up to 1 mM and positive at 10–50 mM	[21]
Benzyl alcohol (C_6_H_5_CH_2_OH)	+	nd	0.1–50 mM, lack of genotoxicity or inconclusive up to 25 mM and positive at 50 mM	[21]
Benzoic acid (C_6_H_5_COOH)	+	nd	0.1–50 mM, lack of genotoxicity or inconclusive up to 25 mM and positive at 50 mM	[21]
Butylparaben (C_11_H_14_O_3_)	-	nd	100–250 mM, lack genotoxicity up to 150 mM and inconclusive up to 250 mM	[22]
Ethylparaben (C_9_H_10_O_3_)	-	nd	100–250 mM, lack genotoxicity up to 200 mM and inconclusive at 250 mM	[22]
Potassium nitrite (KNO_2_)	+	nd	25–100 mM, KNO_2_ lacks genotoxicity at 25 mM	[23]
Potassium nitrate (KNO_3_)	+	nd	25–100 mM, KNO_3_ lacks genotoxicity at 25 mM	[23]
Sodium nitrite (NaNO_2_)	+	nd	25–100 mM, NaNO_2_ lacks genotoxicity at 25 mM	[23]
Sodium nitrate (NaNO_3_)	+	nd	25–100 mM, NaNO_3_ lacks genotoxicity at 25 mM	[23]
**Food dyes**				
Amaranth (C_20_H_11_N_2_Na_3_O_10_S_3_)	+	nd	1–50 mg/mL, inconclusive at 1 mg/mL and positive at 12.5–50 mg/mL	[24]
Carminic acid (C_22_H_20_O_13_)	-	nd	1–20 mg/mL, lack of genotoxicity up to 10 mg/mL and inconclusive at 20 mg/mL	[24]
Erythrosine (C_20_H_6_I_4_Na_2_O_5_)	-	nd	1–6 mg/mL, lack of genotoxicity up to 3 mg/mL and inconclusive at 6 mg/mL	[24]
Indigotine (C_16_H_8_N_2_Na_2_O_8_S_2_)	-	nd	0.25–1 mg/mL, lack of genotoxicity up to 1 mg/mL	[24]
Patent Blue (C_27_H_31_N_2_O_7_S_2_ · 0.5Ca)	+	nd	6.25–25 mg/mL, inconclusive up to 12.5 mg/mL and positive at 25 mg/mL	[24]
**Food flavors**				
L-Carveol (C_10_H_16_O)	-	nd	1.5–5 µL/mL, all doses lack genotoxicity	[25]
(−)-Carvyl acetate (C_12_H_18_O_2_)	-	nd	1.5–5 µL/mL, all doses lack genotoxicity	[25]
(+)-Dihydrocarvone (C_10_H_16_O)	-	nd	1.5–5 µL/mL, all doses lack genotoxicity	[25]
Dihydrocarveol (C_10_H_18_O)	-	nd	1.5 and 2.5 µL/mL, all doses lack genotoxicity	[25]
(−)-Fenchone (C_10_H_16_O)	-	nd	1.5–5 µL/mL, all doses lack genotoxicity	[25]
*S*-(−)-Limonene (C_10_H_16_)	-	nd	1.5–5 µL/mL, 1.5, and 2.5 µL/mL lack genotoxicity and inconclusive at 5 µL/mL	[25]
(±)-Linalool	-	nd	1.5–10 µL/mL, lack of genotoxicity up to 2.5 µL/mL and inconclusive at 10 µL/mL	[25]
α-Phellandrene (C_10_H_16_)	+	nd	1.5–10 µL/mL, all doses show genotoxicity	[25]
**Food products**				
Fresh Inca peanut seed	nd	-	145 mg/mL, lack of genotoxicity	[26]
Heated virgin olive oil	-	nd	6 and 12% *v*/*v*, all doses lack genotoxicity	[27]
Honey-sweetened cashew- apple nectar	-	-	12.5–100% *v*/*v*, all doses lack genotoxicity	[28]
Red pear tomato	-	nd	0.625 and 5 mg/mL, all doses lack genotoxicity	[29]
Soybean oil	+	nd	6, 12, and 24% *v*/*v*, all doses show genotoxicity	[30]
Sunflower oil	-	nd	6, 12, and 24% *v*/*v*, only 12% *v*/*v* shows genotoxicity	[30]
Unheated virgin olive oil	-	nd	6 and 12% *v*/*v*, all doses lack genotoxicity	[27]
**Plant/herbal extracts**				
*Anoectochilus burmannicus*, (hot water extract)	nd	-	500 µg/mL, lack of genotoxicity	[31]
*Anoectochilus burmannicus* (ethanolic extract)	nd	-	2 and 4 mg/mL, all doses lack genotoxicity	[32]
*Artemisia herba-alba* (ethanolic extract)	-	nd	0.5, 1, and 1.5% *v*/*v*, all doses lack genotoxicity	[33]
*Cryptocarya alba* Mol. (aqueous extract)	-	nd	4.74, 9.49, and 18.99 mg/mL, all doses lack genotoxicity	[34]
*Equisetum myriochaetum*	-	-	0.78–500 µg/mL, all doses lack genotoxicity in both crosses	[35]
*Lawsonia inermis* Linn.	nd	-	500 and 1000 µg/mL, all doses lack genotoxicity	[36]
*Mangifera indica* Linn. (aqueous extract)	-	-	1.25, 2.5, and 5 mg/mL, all doses lack genotoxicity in both crosses	[37]
*Peumus boldus* Mol. (aqueous extract)	-	nd	4.56, 9.12, and 18.25 mg/mL, all doses lack genotoxicity	[34]
*Turnera subulate* (aqueous extract)	+	-	5, 10, and 20 mg/mL, all doses show genotoxicity in ST cross; however, HB cross shows inconclusive results	[38]
**Phytochemicals**				
Apigenin	-	nd	0.46 and 1.85 mM, all doses lack genotoxicity	[39]
Betulinic acid	-	-	0.0312–0.5 mM, all doses lack genotoxicity in both crosses	[40]
Bisabolol	-	nd	0.56 and 2.24 mM, all doses lack genotoxicity	[39]
Isoeugenol	-	-	1–15 mM, all doses lack genotoxicity in both crosses	[15]
Lapachol	-	+	20, 40, and 60 µg/mL, all doses lack genotoxicity in ST cross; however, all doses show genotoxicity in HB cross	[41]
Lycopene	-	nd	7 and 56 µM, all doses lack genotoxicity	[29]
Protocatechuic acid	-	nd	0.25 and 1 mM, all doses lack genotoxicity	[39]
Safrole	-	+	0.125–0.75 mM, all doses lack genotoxicity in ST cross; however, all doses show genotoxicity in HB cross	[15]
Vitexin	-	-	0.15, 0.3, and 0.6 mM, all doses lack genotoxicity	[42]

nd—not determined, (-)—negative, and (+)—positive.

**Table 2:** Applications of SMART for testing some medicines.

Types	Result in ST Cross	Result in HB Cross	Range of Treated Doses and Remarks	Ref.
Atorvastatin	-	-	0–340 µM, all doses lack genotoxicity both ST and HB cross	[49]
Bupivacaine	-	-	10–500 µg/mL, inconclusive up to 500 µg/mL in ST cross and HB cross, inconclusive at 10 and 100 but lack genotoxicity at 250 and 500 µg/mL	[50]
Bupropion hydrochloride	+	+	0.937–7.5 mg/mL, inconclusive at 0.937 mg/mL and positive at 1.875–7.5 mg/mL in ST cross, whereby all of doses positive in HB cross	[51]
Doxorubicin	+	+	0.125 µM a dose show positive both ST and HB cross	[49]
Glimepiride	+	nd	10–100 µg, inconclusive at 10 µg and positive at 25–100 µg	[44]
Glipizide	+	nd	10–100 µg, inconclusive at up to 10 µg and positive at 25–100 µg	[44]
Levobupivacaine	-	+	100–1000 µg/mL, inconclusive up to 1000 µg/mL in ST cross; however, inconclusive up to 500 and positive at 1000 µg/mL in HB cross	[50]
Rosuvastatin	-	-	0–300 µM, all doses lack genotoxicity both ST and HB cross	[49]
Trazodone hydrochloride	+	+	0.937–7.5 mg/mL, all doses positive both ST and HB cross	[51]

nd—not determined, (-)—negative, and (+)—positive.

**Table 3:** Applications of SMART for testing insecticide and herbicides.

Types	Result in ST Cross	Result in HB Cross	Range of Treated Doses and Remarks	Ref.
**Insecticides**				
Fipronil	+	+	0.3 × 10^−5^–3.0 × 10^−5^ mM, all of doses positive in ST and HB cross; however, inconclusive at 0.7 × 10^−5^ mM in ST cross	[62]
Metofluthrin	+	nd	6–60 ug/mL, lack of genotoxicity at 6 µg/mL and positive at 60 µg/mL	[70]
Thiamethoxam	-	+	2.4 × 10^−4^–1.9 × 10^−3^ mM, all doses lack genotoxicity; however, 9.7 × 10^−4^–1.9 × 10^−3^ mM were positive in HB cross.	[56]
Transfluthrin	+	nd	0.0103–0.103 mg/mL, positive at all tested doses	[70]
**Herbicides**				
2,4,5-Trichlorophenoxyacetic acid (2,4,5-T)	-	-	0.05–10 mM, lack of genotoxicity up to 0.1 mM and inconclusive at 0.5–10 mM in ST cross; however, lack of genotoxicity at 0.05, inconclusive at 0.1–5 mM in HB cross	[71]
Amitrole	+	nd	0.05–1 mM, inconclusive at 0.1 mM and positive at 0.5–1 mM	[72]
Bentazone	-	+	0.05–10 mM, all doses inconclusive in ST cross; however, all doses positive in HB cross	[73]
Diquat dibromide	-	nd	1–10 mM, all doses inconclusive	[72]
Glyphosate	+	-	0.1–10 mM, inconclusive up to 1 mM and positive at 2–10 mM in ST cross, whereby lack of genotoxicity at 0.1–0.5 and 2–5 mM, inconclusive at 1 and 10 mM in HB cross	[71]
Hydrazide	+	+	0.1–10 mM, inconclusive up to 0.5 mM and positive 1–10 mM in ST cross; HB cross shows lack of genotoxicity up to 0.5 mM, inconclusive at 1–5 mM and positive at 10 mM in HB cross	[71]
Imazamox	+	-	2.5–20 mM, lack of genotoxicity at 2.5–5 mM, and weak positive at 10–20 mM in ST cross; however, all doses inconclusive both HB cross	[74]
Imazapic	-	-	2.5–20 mM, all doses lack genotoxicity in ST and HB cross	[74]
Imazethapyr	-	-	2.5–20 mM, all doses lack genotoxicity in ST and HB cross	[74]
Metribuzin	-	nd	1–10 mM, lack of genotoxicity at 5 mM and inconclusive at 1, 2, and 10 mM	[72]
Molinate	+	+	0.1–10 mM, inconclusive up to 1 mM and positive at 2–10 mM in ST cross; moreover, inconclusive up to 2 mM and positive at 5–10 mM in HB cross	[72]
Prometryn	-	nd	1–10 mM, all doses inconclusive	[72]
Propanil	+	+	0.1–10 mM, lack of genotoxicity at 0.1 mM, inconclusive at 0.5–5 mM, and positive 10 mM in ST cross; however, inconclusive at 0.1 mM and positive at 0.5–10 mM in HB cross	[71]
Terbutryn	+	nd	1–10 mM, inconclusive at 1, 2, and 10 mM and positive at 5 mM	[72]
Thiobencarb	+	-	0.1–10 mM, inconclusive up to 5 mM and positive at 10 mM in ST cross; all doses lack genotoxicity in HB cross	[73]
Trifluralin	+	+	0.1–10 mM, ST cross shows inconclusive up to 5 mM and positive at 10 mM; however, inconclusive at 0.1 mM and positive at 0.5–10 mM in HB cross	[73]

nd—not determined, (-)—negative, and (+)—positive.

**Table 5:** Applications of SMART for testing wastewater.

Types	Result in ST Cross	Result in HB Cross	Range of Treated Doses and Remarks	Ref.
Water samples from Candiota Stream (Candiota, Brazil) in summer	-	-	Collected from four locations, all locations lack genotoxicity both ST and HB cross	[98]
Water samples from Candiota Stream (Candiota, Brazil) in winter	-	-	Collected from four locations, all locations lack genotoxicity both ST and HB cross	[98]
Water samples from Mumbuca stream and Perdizes river, Brazil	-	-	Collected from five locations, all locations lack genotoxicity both ST and HB cross	[99]
Water samples from Caı’ river, Brazil, in March	+	-	Collected from three locations, all locations positive genotoxicity in ST; however, collected from two locations, all locations lack mutagenicity in HB cross	[100]
Water samples from Caı´ river, Brazil, in June	-	-	Collected from three locations, most samples exhibited lack of mutagenicity or inconclusive	[100]
Water samples from Caı´ river, Brazil, in September	-	-	Collected from three locations, most samples exhibited lack of mutagenicity or inconclusive	[100]
Water samples from Córrego do Óleo and Córrego Liso	+	+	All samples of water showed genotoxicity in ST and HB cross	[97]
Water samples of Sinos river and Araçá and Garças Streams (Canoas, Brazil)	-	-	All samples lack genotoxicity both ST and HB cross	[101]

(-)—negative, and (+)—positive.

**Table 6:** Applications of SMART for testing nanoparticles.

Types	Result in ST Cross	Result in HB Cross	Range of Treated Doses and Remarks	Ref.
Carbon nanotubes	-	nd	64–1000 µg/mL, all doses lack genotoxicity	[110]
Carbon nanotubes	-	-	50–250 µg/mL, lack genotoxicity at 50, 100, and 200 µg/mL and inconclusive at 150 and 250 µg/mL in both ST and HB cross	[111]
Cobalt nanoparticles	-	nd	0.1–10 mM, lack genotoxicity up to 5 mM and inconclusive at 10 mM	[112]
Copper oxide nanoparticles (CuONPs)	+	nd	0.24–0.95 mg/mL, lack genotoxicity at 0.24 mg/mL and inconclusive at 0.48 mg/mL but positive at 0.95 mg/mL in ST cross	[105]
Gold nanoparticles	-	-	20–30 µg/mL, all of doses inconclusive in ST cross, whereby all of concentrations lack genotoxicity in HB cross	[113]
Iron oxide nanoparticle (<50 nm)	+	nd	1–10 mM, inconclusive at 2 and 5 mM, positive at 1 and 10 mM	[114]
Iron oxide nanoparticle (<100 nm)	-	nd	1–10 mM, inconclusive at 1 mM and lack genotoxicity 2–10 mM	[114]
Iron nanoparticles	-	nd	0.1–10 mM, all concentrations lack genotoxicity	[115]
Nickel oxide nanoparticles	+	+	1.31–21 mg/mL, all of doses positive in ST cross but lack genotoxicity up to 10.50 mg/mL and positive at 21 mg/mL in HB cross	[108]
Titanium dioxide nanocrystal (A3.4 TiO_2_ NCs)	+	+	1.5625–12.5 mM, lack genotoxicity up to 6.25 and positive at 12.5 mM in ST cross; moreover, at 12.5 mM lack genotoxicity, 6.25 mM inconclusive, and positive at 1.5625 and 3.125 mM in HB cross	[112]
Titanium dioxide nanocrystal (A6.2 TiO_2_ NCs)	-	-	1.5625–12.5 mM, lack genotoxicity up to 12.5 and inconclusive at 1.5625 in both ST and HB cross	[112]
Titanium dioxide nanoparticles	-	nd	0.08–1.60 mg/mL, all concentrations lack genotoxicity	[106]
Zinc oxide nanoparticles	+	-	0.075–1.2 mg/mL, lack genotoxicity up to 0.15 mg/mL and positive at 0.3–1.2 mg/mL in ST cross; however, all of doses lack genotoxicity in HB cross	[116]

nd—not determined, (-)—negative, and (+)—positive.

**Table 7:** The roles of some phytochemicals or herbal extracts against known mutagens/carcinogens using SMART assay.

Genotoxins	Tested Compounds/Extracts	Anti-Genotoxic Properties	Results and Remarks
**Benzo[a]pyrene (BaP)**			
	Vitexin	Yes	0.15, 0.3, and 0.6 mM vitexin inhibit wing spot formation induced by 1 mM BaP [42]
**Doxorubicin (DXR)**			
	*Hymenaea courbaril* extract	Yes,	0.3–3 mL of *Hymenaea courbaril* inhibits wing spot formation induced by 0.125 mg/mL DXR [122]
	Noni fruit juice	Yes	25–75% *v*/*v* of noni fruit juice inhibits wing spot formation induced by 0.2 mM DXR [123]
	Propolis (aqueous extract)	Yes	12.5–50 mg/mL of propolis(aqueous extract) inhibits wing spot formation induced by 0.125 mg/mL DXR [124]
**Ethyl methanesulfonate (EMS)**			
	Boron	Yes	0.1–40 mg/mL of boron inhibits wing spot formation induced by 0.1 mM EMS [125]
	*Buddleja globosa* leaf extract	Yes	1.91, 3.83, and 7.66 mg/mL of *Buddleja globosa* inhibit wing spot formation induced by 0.12 mg/mL EMS [121]
	*Citrus aurentium* peel oil	Yes	0.1–0.5% *v*/*v* of *Citrus aurentium* fruit peel oil inhibits wing spot formation induced by 0.5 mM EMS [126]
	*Cryptocarya alba* leaf extract	Yes	4.74–9.79 mg/mL of *Cryptocarya alba* inhibits wing spot formation induced by 0.12 mg/mL EMS [34]
	*Peumus boldus* leaf extract	Yes	2.28–9.12 mg/mL of *Peumus boldus* extract inhibits wing spot formation induced by 0.12 mg/mL EMS [34]
**Hydrogen peroxide (H_2_O_2_)**			
	*Brassica carinata* leaf extract	Yes	1.25–5.0 mg/mL of *Brassica carinata* extract inhibits wing spot formation induced by 0.12 M H_2_O_2_ [127]
	Sinigrin	Yes	0.6–4.81 mM sinigrin, a glucosinolate compound, inhibits wing spot formation induced by 0.12 M H_2_O_2_ [127]
	Red pear tomato	Yes	0.625 and 5 mg/mL of red pear tomato extract inhibit wing spot formation induced by 0.12 M H_2_O_2_ [29]
	Lycopene	Yes	7 and 56 µM lycopene inhibit wing spot formation induced by 0.12 M H_2_O_2_ [29]
	Lemon juice	Inconclusive	0.75 and 50% *v*/*v* lemon juice showed inconclusive inhibitory effect induced by 0.15 M H_2_O_2_ [128]
	Orange juice	Inconclusive	0.75 and 50% *v*/*v* lemon juice showed inconclusive inhibitory effect induced by 0.15 M H_2_O_2_ [128]
	Hesperidin	Inconclusive	0.0038 and 0.24 mM of hesperidin showed inconclusive inhibitory effect induced by 0.15 M H_2_O_2_ [128]
	Limonene	Inconclusive	0.011 and 0.73 mM of limonene showed inconclusive inhibitory effect induced by 0.15 M H_2_O_2_ [128]
**Mitomycin C (MMC)**			
	Chalcone	Yes	10–400 µg/mL of chalcone inhibits wing spot formation induced by 0.05 mM MMC [129]
	Coumarin–chalcone hybrid	Yes	5–400 µg/mL of coumarin–chalcone hybrid inhibits wing spot formation induced by 0.05 mM MMC [129]
**N-nitroso N-ethylurea (ENU)**			
	Ascorbic acid	Yes	17 mM Ascorbic acid inhibits wing spot formation induced by 0.01 mM ENU [130]
	*Citrus aurentium* peel oil	Yes	0.1–0.5% *v*/*v* of *Citrus aurentium* fruit peel oil inhibits wing spot formation induced by 0.01 mM ENU [126]
**Urethane (URE)**			
	Betulinic acid	Yes	1.64, 3.28, and 6.57 mM of betulinic acid inhibit wing spot formation induced by 10 mM URE [131]
	*Origanum Compactum* essential oil	Yes	0.05 and 0.1% *v*/*v* of *Origanum Compactum* essential oil inhibits wing spot formation induced by 10 mM URE [132]
	*Ficus dubia* latex	No	0.25, 1, and 2 mg/mL of *Ficus dubia* latex did not inhibit wing spot formation induced by 20 mM URE [133]
	*Ficus dubia* root extract	Weak	0.25, 1, and 2 mg/mL of *Ficus dubia* root extract weakly inhibits wing spot formation induced by 20 mM URE [133]

Benzo[a]pyrene (BaP) is a well-known promutagen presented in the environment and in grilled or smoked food. BaP metabolizes into benzo(a)pyrene-7,8-diol 9,10-epoxide (BPDE), derived from phase I and Phase II enzymes that arrest and impede the progress of the DNA replication fork [134] and are highly mutagenic and carcinogenic [135]. Doxorubicin (DXR) is an antineoplastic drug that inhibits DNA and RNA synthesis as well as constraining topoisomerase II [136]. Ethyl methanesulfonate (EMS) is an ethylating agent that causes DNA mutations by transferring alkyl groups to nucleotides, finally leading to chromosomal aberrations [137,138]. Hydrogen peroxide is an oxidative mutagen that induces microsatellite instability in mismatch repair-deficient *D. melanogaster* [139]. Mitomycin C (MMC) is a chemotherapeutic DNA cross-linking agent resulting in alkylated guanines [140]. N-nitroso N-ethylurea (ENU) is a direct-acting mutagen/carcinogen produced mainly by GC-AT transition via ethylation and carbamoylation [141]. Urethane (URE) or ethyl carbamate is a carcinogen found in fermented food and beverage. URE undergoes biotransformation in the liver, resulting in vinyl carbamate epoxide that is reactive to DNA and RNA [142].

## Data Availability

Not applicable.

## Abbreviations

2,4,5-T	2,4,5-Trichlorophenoxyacetic acid
2-MIB	2-methylisoborneol
BaP	Benzo[a]pyrene
BPA	bisphenol A
BPDE	Benzo(a)pyrene-7,8-diol 9,10-Epoxide
Cd	cadmium
CNTs	carbon nanotubes
Co-NPs	cobalt nanoparticles
CuO NPs	copper oxide nanoparticles
DWCNTs	double-walled carbon nanotubes
DXR	Doxorubicin
EFSA	European Food Safety Authority
EMS	Ethyl methanesulfonate
ENU	N-nitroso N-ethylurea
H_2_O_2_	Hydrogen peroxide
HB cross	High bioactivation cross
LOH	Loss of heterozygosity
MMC	Mitomycin C
NaNO_2_	Sodium nitrite
NaNO_3_	Sodium nitrate
NCDs	non-communicable diseases
NiO-NPs	nickel-based nanoparticles
NNK	4-(methylnitrosamino)-1-(3-pyridyl)-1-butanone
NNN	N-Nitrosonornicotine
NOCs	N-nitroso compounds
PAHs	polycyclic aromatic hydrocarbons
ROS	Reactive oxygen species
SMART	Somatic mutation and recombination test
SSRIs	selective serotonin reuptake inhibitors
ST cross	Standard cross
SWNTs	single-walled carbon nanotubes
T2DM	type 2 diabetes mellitus
TiO2 NCs	Titanium dioxide nanocrystals
URE	Urethane
